# Mechanical fingerprints in breast cancer research: A multimodal experimental approach

**DOI:** 10.1002/btm2.70148

**Published:** 2026-05-08

**Authors:** Federica Banche‐Niclot, Rosalia Ferraro, Valerio Di Palo, Paola de Paolis, Francesca Taraballi, Sergio Caserta

**Affiliations:** ^1^ Center for Musculoskeletal Regeneration Houston Methodist Academic Institute Houston Texas USA; ^2^ Orthopedics and Sports Medicine Houston Methodist Hospital Houston Texas USA; ^3^ Department of Chemical, Material and Industrial Production Engineering University of Naples “Federico II” Naples Italy; ^4^ CEINGE Advanced Biotechnologies Franco Salvatore Naples Italy

**Keywords:** 3D spheroid models, breast cancer, tissue stiffness, translational cancer models, tumor biomechanics, viscoelastic properties

## Abstract

Breast cancer remains the leading cause of cancer‐related mortality among women worldwide. Tumor biomechanics are not merely a symptom: they represent a functional signature with translational relevance in diagnostic, prognostic, and therapeutic resistance. Despite this, few experimental models are engineered to systematically investigate these physical properties across biological systems. Here, this study presents a multimodal biomechanical platform combining engineered 3D breast cancer spheroids with *ex vivo* tissue analysis to profile and compare viscoelastic behavior of healthy and tumoral environments. Rheometry and compression testing revealed a consistent mechanical shift in tumor‐derived samples marked by increased stiffness and force‐dependent nonlinear behavior, mirroring the ECM remodeling typical of aggressive phenotypes. This increased rigidity may adversely affect chemotherapy effectiveness by hindering drug delivery and altering cellular mechanotransduction. These biomechanical fingerprints enable quantitative discrimination between healthy and cancerous tissues and can serve as a surrogate marker of malignancy. By supporting the development of mechanics‐informed diagnostic tools, our platform offers a reproducible, clinically relevant framework to integrate biomechanical screening into translational breast cancer pipelines.

## Graphical abstract

Multimodal biomechanical platform integrating *in vitro* 3D spheroids and *ex vivo* tissues to compare healthy and tumor viscoelastic properties. Rheological measurements and compression tests reveal increased stiffness and pronounced nonlinear mechanical behavior in tumor samples, highlighting distinct biomechanical signatures associated with cancer progression and microenvironmental remodeling.

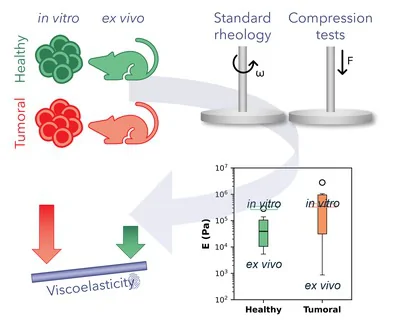

**Translational Impact Statement**

We propose a multimodal experimental approach that combines *in vitro* 3D breast‐cancer models and *ex vivo* tissue analysis to measure and compare the viscoelastic properties of healthy and malignant breast tissues. By using mechanical behavior as a fingerprint, this framework discriminates tumor tissue from its healthy counterpart. Revealing how tumor stiffness impacts drug delivery and therapy resistance, the approach provides a clinically relevant tool to inform diagnosis and optimize treatment strategies.

## 1 INTRODUCTION

Breast cancer is the most frequently diagnosed malignancy and a leading cause of cancer‐related mortality among women worldwide.^1^ While survival rates have improved for localized tumors thanks to early detection and localized treatments (such as surgery and targeted chemotherapy), metastatic breast cancer remains the leading cause of death, accounting for over 90% of fatalities.^2^ This highlights the critical and urgent need for models that capture molecular features along with the physical complexity of the disease.

In recent years, tumor biomechanics have emerged as a critical dimension of cancer biology. Mechanical alterations within the tumor microenvironment, including extracellular matrix (ECM) stiffness, altered tissue viscoelasticity, and increased cellular deformability,^3^ are not passive byproducts of disease. Rather, increasing evidence shows that such biomechanical cues play an active role in reshaping cellular behavior and microenvironmental interactions,^4^ by actively influencing key hallmarks of cancer: invasive and migrate, survive under stress, and respond to chemotherapeutic agents.^5^, ^6^ Tumor tissues often exhibit altered viscoelasticity and reduced compressibility, while cancer cells adapt through cytoskeletal remodeling and mechanical plasticity.^7^, ^8^

Despite this, mechanical phenotype remains underutilized in current diagnostic and therapeutic workflows and few platforms allow for controlled and quantitative exploration of these mechanical dynamics.^9^ Conventional 2D cultures lack the spatial,^10^, ^11^, ^12^, ^13^ heterogenicity^14^, ^15^ and mechanical context^16^ of real tissues; while animal models offer limited tunability, scalability and translational accuracy.^17^ 3D tumor spheroids (*i.e*., multicellular compact aggregates) are gaining increased interest as a promising bridge, capable of recapturing the architecture, gradients, and cell‐ECM interactions of native tumors as a scalable platform.^18^, ^19^ However, their biomechanical characterization remains challenging and underexplored. *Ex vivo* experiments, which involves tissues or organs from living organisms, can come into play allowing also the maintenance of tissue natural architectures and cell‐to‐cell interactions, minimizing alteration to the living environment and thus offering a more realistic approximation of *in vivo* conditions.^20^ Nevertheless, a clinically relevant *in vitro* model must be quantitatively validated against real tissue mechanics using reliable and physiological meaningful techniques.

Traditional techniques such as atomic force microscopy (AFM),^21^, ^22^, ^23^ indentation,^24^ micro tweezers,^25^ and compression assay^26^, ^27^ have contributed valuable insights into cellular elasticity, yet they often oversimplify the inherently viscoelastic nature of biological systems. These methods are sensitive to tool geometry and substrate properties, resulting in high variability and limited comparability especially when transitioning from isolated cells to tissue‐level constructs. In contrast, rheology provides a robust and scalable method to quantify viscoelastic behavior in both soft solids and complex biological matrices. By measuring elastic (*G*′) and viscous (*G*″) moduli, rheology identifies both energy storage and dissipation, which are key parameters for understanding how tumors deform, resist, or transmit mechanical stress. These moduli can be translated into conventional mechanical metrics (*e.g*., Young's modulus) under defined assumptions, allowing comparisons across biological models.^28^ Given the deformability, heterogeneity, and flow‐like behavior of cancerous tissues, rheological analysis offers a physiologically relevant and reproducible approach for characterizing tumor mechanics at experimental scales.

In this study, we present a multimodal experimental strategy that couples *in vitro* spheroids with *ex vivo* tissue analysis to quantify the viscoelastic profiles of healthy and cancerous breast environments. Using rotational rheometry and compression testing, we mapped the viscoelastic landscape of breast cancer and healthy controls.

Our approach aims to establish a reproducible mechanical benchmark that distinguishes mechanical alterations associated with tumor pathology, laying the foundation for incorporating biomechanics into translational breast cancer research with potential application in both diagnostic development and therapy optimization.

## 2 MATERIALS AND METHODS

### 2.1 Cell culture and spheroids formation

The biological component of this study relied on two distinct cell lines, organized into spheroids to better replicate the three‐dimensional tumor microenvironment. The first model system consisted of MDA‐MB‐231 human breast adenocarcinoma cells (official designation: MDA‐MB‐231, HTB‐26; Homo sapiens, female; pleural effusion from a patient with metastatic breast adenocarcinoma; Research Resource Identifier (RRID):CVCL_0062), obtained from the American Type Culture Collection (ATCC, Manassas, VA, USA) in March 2022. ATCC authenticates this cell line by short tandem repeat profiling, and MDA‐MB‐231 is not listed as misidentified or cross‐contaminated in the International Cell Line Authentication Committee register.

As a control, primary human mesenchymal stem cells (FP‐MSCs) were isolated from infrapatellar fat pad tissue (male donor, 24 years old, height 188 cm, weight 72.6 kg, BMI 20.5) under institutionally approved protocols (Houston Methodist Hospital IRB Pro00015718) in November 2022. FP‐MSCs were characterized by the institutional biorepository using standard MSC immunophenotyping (CD73+, CD90+, CD105+, CD45−) prior to experimental use. Because FP‐MSCs are primary cells rather than commercial or immortalized lines, a RRID is not applicable.

Mycoplasma testing was not performed on either MDA‐MB‐231 or FP‐MSC during this study. While this represents a potential limitation, no morphological abnormalities or altered growth kinetics were observed, and the reproducibility of biomechanical results across replicates supports that contamination did not influence the experimental outcomes.

The MDA‐MB‐231 were cultured in Dulbecco's Modified Eagle's Medium (DMEM) supplemented with 20% (v/v) fetal bovine serum (FBS) and 1% (v/v) antibiotics (50 U/mL penicillin and 50 mg/mL streptomycin). Cells were used at passages 3–9. On the other hand, FP‐MSC was obtained from the orthopedic biorepository at Houston Methodist Hospital, which were previously obtained by the Department of Orthopedics and Sports Medicine Department (IRB Pro00015718). Those were cultured in alpha minimum essential medium supplemented with 20% (v/v) FBS and 1% (v/v) antibiotics. Cells were used at passages 1–6. Both cell lines were cultured under sterile standard conditions (37°C, 5% CO_2_). Transiting from 2D to 3D cultures, cellular spheroids were prepared using the liquid overlay technique,^29^ in which a non‐adhesive concave surface was employed to promote cell–cell aggregation within the meniscus. Specifically, the nonstick polymer film on the wells was obtained by coating U‐bottom shaped 96‐well plate (Corning TM Costar TM 96‐Well, Cell Culture‐Treated, U‐Shaped Bottom Microplate, Thermo Fisher Scientific) with 0.1% polyHEMA in 96% ethanol. Cells were then seeded at a concentration of 2 × 10^3^ cells/well. FP‐MSC spheroids were cultured in the medium described above, whereas MDA‐MB‐231‐GFP ones were maintained in an optimized medium (DMEM with 1% antibiotics, 2% bovine serum albumin, 0.05% insulin, and 1% Matrigel). Additionally, the cancer multiwell plate was placed on a rotating plate to enhance the cells aggregation. Both spheroids were then incubated under typical cell culture conditions to obtain compact spheroids of adequate size: 1 day for FP‐MSCs and 3 days for MDA‐MB‐231‐GFPs.

### 2.2 Cell viability assessment

Two complementary assays were employed to evaluate spheroids viability over time. Specifically, at designated time points (24, 48, and 72 h), the ATP‐related metabolic activity of spheroids was quantified through CellTiter‐Glo® 3D Assay (Promega Corporation, Madison, WI, USA) following the manufacturer's instructions. Alongside, live and dead cells were determined via Live/Dead fluorescent staining by using two fluorescence probes that simultaneously color intracellular esterase activity and plasma membrane integrity, recognized parameters of cell viability. For this purpose, cell‐permeant calcein AM (1 μM, Thermo Fisher Scientific) and ethidium homodimer‐1 (EthD‐1, 2 μM, Thermo Fisher Scientific) were used to stain live cells (green fluorescence) and dead cells (red fluorescence), respectively. Samples were incubated with the staining solution for 15 min before imaging. Experiments were performed in triplicate.

### 2.3 Collagenous supporting hydrogel preparation

Collagen‐based support with suitable stiffness was developed to stabilize spheroids during rheological analysis and thus prevent uncontrolled movement and ensure an adequate gap for accurate measurements. For this aim, a purified methacrylated Type I bovine collagen (PhotoCol® Methacrylated Collagen, CMA, Advanced BioMatrix) solution was prepared at a concentration of 3 mg/mL following the manufacturer's instructions. To produce a uniform collagen matrix for supporting spheroids during rheological characterization, 2 mL of collagen solution was poured into a circular silicon mold of 25 mm diameter and a thickness of 2.5 mm, matching the size and shape of the measurement tool. The samples were then incubated at 37°C with 5% CO_2_ for 1 h to ensure physical crosslinking of collagen.

### 2.4 Assembly of collagen‐spheroid constructs

Cellular spheroids were placed on the crosslinked collagen disk before rheological characterization. The number of spheroids was selected to achieve approximately 1% surface coverage (*S*%) of the supporting disk, defined as $n\cdot \left(A_{s}/A_{\mathrm{gel}}\right)\cdot 100=n\cdot {\left(d_{s}/d_{\mathrm{gel}}\right)}^{2}\cdot 100$. Here, *n* is the number of spheroids, *d*_*s*_ the mean spheroids diameter for each cell lines, and *d*_gel_ the diameter of the collagenous hydrogel (COL) (25 mm). The average diameter of the MDA‐MB‐231‐GFP spheroids was calculated to 580.00 ± 30.55 μm, while 256.66 ± 14.53 μm for FP‐MSC spheroids (Figure S1). Based on these values, 15 spheroids were required for MDA‐MB‐231‐GFP cells, whereas 85 spheroids were needed for FP‐MSC cells. Therefore, spheroids were individually collected from the 96‐well plates and dropwise on the hydrogel's surface, ensuring a uniform distribution. Due to the high number of FP‐MSC spheroids required, they were arranged in groups of approximately 10 spheroids each along the matrix's surface.

### 2.5 Tissues manipulation

To evaluate the physiological relevance of the developed *in vitro* spheroid‐based 3D models, the biomechanical properties of murine adipose tissue and tumor masses were examined and compared to those of spheroids. Given that animal models remain the primary established system for studying tumor mechanics, this comparison aimed to assess whether the *in vitro* model reliably recapitulates key biomechanical features observed *in vivo*. To this aim, mammary gland fat tissue from mice was analyzed as the *ex vivo* equivalent of FP‐MSC spheroids, while tumors generated by MDA‐MB‐231 cell injections into mice served as a reference for MDA‐MB‐231‐GFP spheroids. The sample tissues were thawed using 1× phosphate‐buffered saline (PBS) and transferred to a Petri dish, where they were left to rehydrate for a minimum of 5 min to ensure optimal restoration of their physiological properties. Prior to testing, the tissues were removed from the PBS, and if necessary, standardized to a 5 mm diameter using a biopsy punch to ensure consistency in sample size and facilitate reproducible mechanical measurement.

### 2.6 Rheological setup

Oscillatory measurements of both *in vitro* and *ex vivo* systems were carried out by using a stress‐controlled rheometer (Anton Paar Physica MCR 301 Instrument) equipped with a smooth parallel plate measuring system with a diameter of 25 mm (measuring plate PP25‐25MM, Anton Paar). For guaranteeing spheroids' viability by mimicking physiological conditions, the temperature of the rheometer's lower plate was kept at 37°C using a Peltier system. Additionally, to prevent water evaporation and to ensure a uniform temperature around the sample, the rheometer plate was enclosed within a chamber to create a controlled environment. The viscoelastic properties of systems were assessed by performing strain (γ) sweep tests, with amplitudes ranging from 0.05% to 1% at a constant angular frequency (*ω*) of 10 rad/s, to identify the linear viscoelastic range. Elastic and viscous moduli (*G*' and *G*", respectively) were measured by varying *ω* within the range 0.01–100 rad/s.

### 2.7 Compression setup

Compression tests were performed on *ex vivo* tissues by using a mechanical testing system (Univert Cell Scale biomaterials testing) equipped with a 1 N load cell. Measurements were performed on both tumor and non‐tumor tissues, which were standardized to a cylindrical shape with a diameter of 5 mm. The mechanical setup was designed to assess the viscoelastic behavior of the tissues, capturing their response to deformation and short‐term recovery under controlled mechanical stress. For achieve this, a single compression cycle of 3 min, followed by a 5‐s recovery period, was applied with a stretch magnitude of 50%, ensuring a standardized assessment of the tissue's mechanical behavior. Mechanical properties of the samples were expressed in terms of Young's modulus (*E*), determined from the slope of the linear correlation between stress (*σ*) and strain (*ε*).^30^, ^31^

### 2.8 Statistical analysis

Cellular assessments were performed in *n* = 3 independent cultures, with technical triplicates measured per sample. Rheological measurements were performed on at least two independent construct types (specifically, COL *n* = 3, COL+NT *n* = 2, COL+T *n* = 7, healthy tissue *n* = 10 and tumoral tissue *n* = 9), while *n* = 8 compression tests for each tissue type were tested. Two‐group comparisons were analyzed through a two‐way ANOVA method using an unpaired two‐tailed Welch's *t*‐test when variances were not assumed equal. A *p*‐value <0.05 was considered statistically significant. Statistical analyses were performed using GraphPad Prism v10. Data are presented as mean ± standard error of the mean (SEM), where SEM is defined as the standard deviation of the population normalized by the square root of the sample size.

## 3 RESULTS

### 3.1 Spheroids viability

To assess the most appropriate spheroids formation protocol, viability of both FP‐MSC and MDA‐MB‐231‐GFP spheroids was evaluated using CellTiter‐Glo® 3D Assay over a 72‐h period. In particular, optical imaging confirmed the morphologies in 2D monolayer and spheroids set‐up (Figure 1a) while Figure 1b reported the metabolic activity of the 2D control compared with spheroids, healthy cells in the upper histogram and the cancer cells in the lower one. From both cell lines, the spheroids' metabolic activity was significantly lower compared to 2D cultures, particularly for the FP‐MSC condition. This reduced viability in spheroids is likely a result of the physical constraints of their architecture and the formation of a necrotic core, which deprives the central cells of nutrients and oxygen. In contrast, 2D controls exhibited a continuous increase over time, reflecting the proliferation of cells in the bidimensional configuration. Conversely, spheroids are characterized by a non‐proliferative nature and their cells progressively consolidating around the core, as demonstrated by the moderate downward trend over time, particularly for FP‐MSC ones. Despite these differences, spheroids result alive after 3 days of culturing and the observed trend can be ascribed to the expansion of the necrotic core, an intrinsic feature of this type of 3D construct,^17^ as demonstrated by Live/Dead staining (Figure 1c).

**FIGURE 1:**
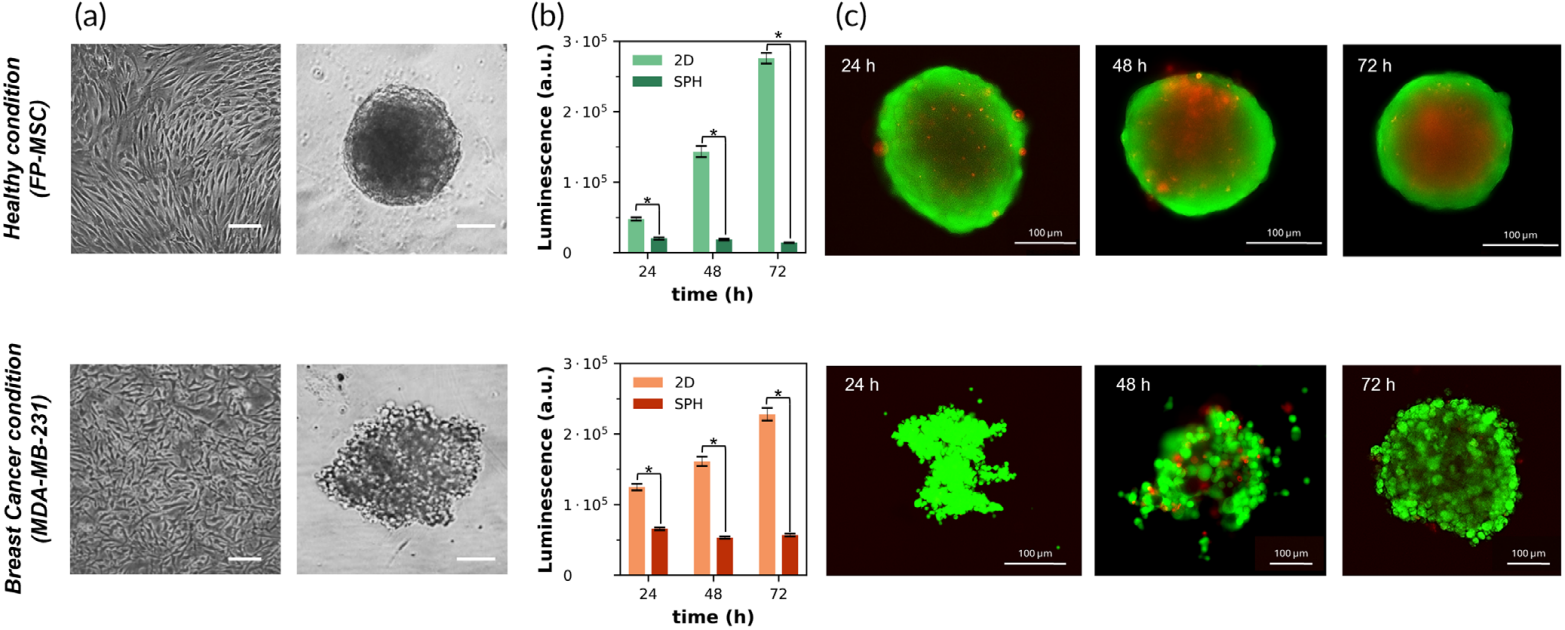
Spheroids morphology and viability investigations: (a) Optical images of 2D monolayer (left) and spheroids (right) for FP‐MSC (above) and MDA‐MB‐231‐GFP (below). Scale bar: 100 μm. (b) Cells viability assessment on both the 2D control and the spheroids configurations at different timepoints (24, 48, and 72 h). Statistical significance of the results: *p <0.05, *n* = 3 for cell type, technical triplicates. (c) Live/Dead images of FP‐MSC spheroids (above) and MDA‐MB‐231‐GFP (below) spheroids at different timepoints (24, 48, and 72 h).

Considering the FP‐MSC spheroids images (Figure 1c, top row), they initially exhibited a compact structure with mixed fluorescence, with green indicating live cells and red indicating dead cells. However, as time progressed, a clear necrotic core began to form, particularly evident after 72 h. This was reflected by the increased red fluorescence, indicating a higher number of non‐viable cells. Given the higher metabolic activity and the absence of a necrotic core, FP‐MSC spheroids were tested after 24 h to ensure their viability.

Different behaviors were observed for MDA‐MB‐231‐GFP spheroids. Initially, the cells are unable to aggregate into a well‐defined 3D structure at 24 and 48 h. A nearly spherical shape morphology was observed only at 72 h. Additionally, the development of a necrotic core moderately starts to develop at the last timepoint. Despite the onset of necrosis, the metabolic activity remained nearly constant over the 72‐h experimental period and was not significantly influenced by the necrotic core. Therefore, due to the sustained viability at 72 h and the absence of a fully developed necrotic core, the mechanical properties of MDA‐MB‐231 spheroids were assessed at this timepoint to ensure proper spheroid formation.

### 3.2 Mechanical characterization of *in vitro* 3D models

Rheological analyses were performed on the *in vitro* 3D models, using collagen‐based hydrogel as supporting material (Figure 2). FP‐MSC and MDA‐MB‐231‐GFP spheroids were embedded in the collagen‐based hydrogel (Figure 2a). Bright field/fluorescence imaging confirmed a uniform spheroid distribution throughout the supporting material (Figure 2b). Samples were then mounted in a plate–plate rheometer for amplitude oscillatory testing (Figure 2c). To ensure a comprehensive evaluation, the viscoelastic properties of the COL were also characterized independently as a reference. Figure 2d presents the rheological outcomes for non‐tumoral (NT‐S, composed of FP‐MSC) and tumoral (T‐S, composed of MDA‐MB‐231‐GFP) spheroids, highlighting key differences in their viscoelastic behavior in terms of *G*' and *G*" (represented by upward and downward triangles, respectively) reported as function of *ω*. As shown, the viscoelasticity properties of pure collagen (data reported in blue) are approximately three orders of magnitude lower than those of the system containing spheroids (COL+NT‐S in green and COL+T‐S in orange, respectively). At *ω*∼1 rad/s, *G*′ for the supporting hydrogel alone is approximately 78 Pa, whereas for the *in vitro* 3D systems, *G*′ reaches 9.70 ± 1.83 × 10^4^ Pa for COL+NT‐S and 2.45 ± 1.13 × 10^5^ Pa for COL+T‐S. This confirms that collagen stiffness does not influence the mechanical properties of the spheroids, making it a suitable support material that mimics the soft ECM environment around the cells. Interestingly, the biomaterial alone exhibits a crossover point (*G*′ = *G*″) at *ω*∼45 rad/s, indicating a transition in its mechanical behavior from solid‐like to liquid‐like. Contrarily, no crossover is observed in the samples containing spheroids, suggesting a persistently dominant elastic response throughout the tested frequency range and reinforcing the solid‐like nature of spheroids. In addition, the incorporation of spheroids enhanced the structural integrity and elastic nature of the construct. This aligns with the behavior of native tissue, which exhibits a predominantly elastic response due to its ECM composition and cell–cell interactions.^32^ Thus, these results highlight the importance of cellular components in maintaining the elastic nature of tissue and confirm that the spheroid‐laden hydrogel more effectively mimics the biomechanical properties, providing a more physiologically relevant *in vitro* model for studying tissue mechanics. Focusing on the *in vitro* 3D models, tumoral spheroids exhibited a higher *G*′ compared to healthy ones (approximately 2.45 × 10^5^ Pa and 9.70 × 10^4^ Pa, respectively, at ω∼1 rad/s). Particularly, the *G*′ values of these spheroids were consistent across collagen concentrations, further suggesting that the mechanical properties of FP‐MSC spheroids are independent of the supporting matrix stiffness within this range.^33^, ^34^ In stark contrast, tumoral MDA‐MB‐231‐GFP spheroids exhibited significantly higher stiffness, with a *G*′ value 2.5 times greater than that of FP‐MSC spheroids, which is consistent with the widely observed phenomenon of tumor tissues being mechanically stiffer than healthy tissues. This increase in stiffness was accompanied by a higher value of tan δ, which is indicative of a more solid‐like behavior in tumor spheroids compared to their healthy counterparts.

**FIGURE 2:**
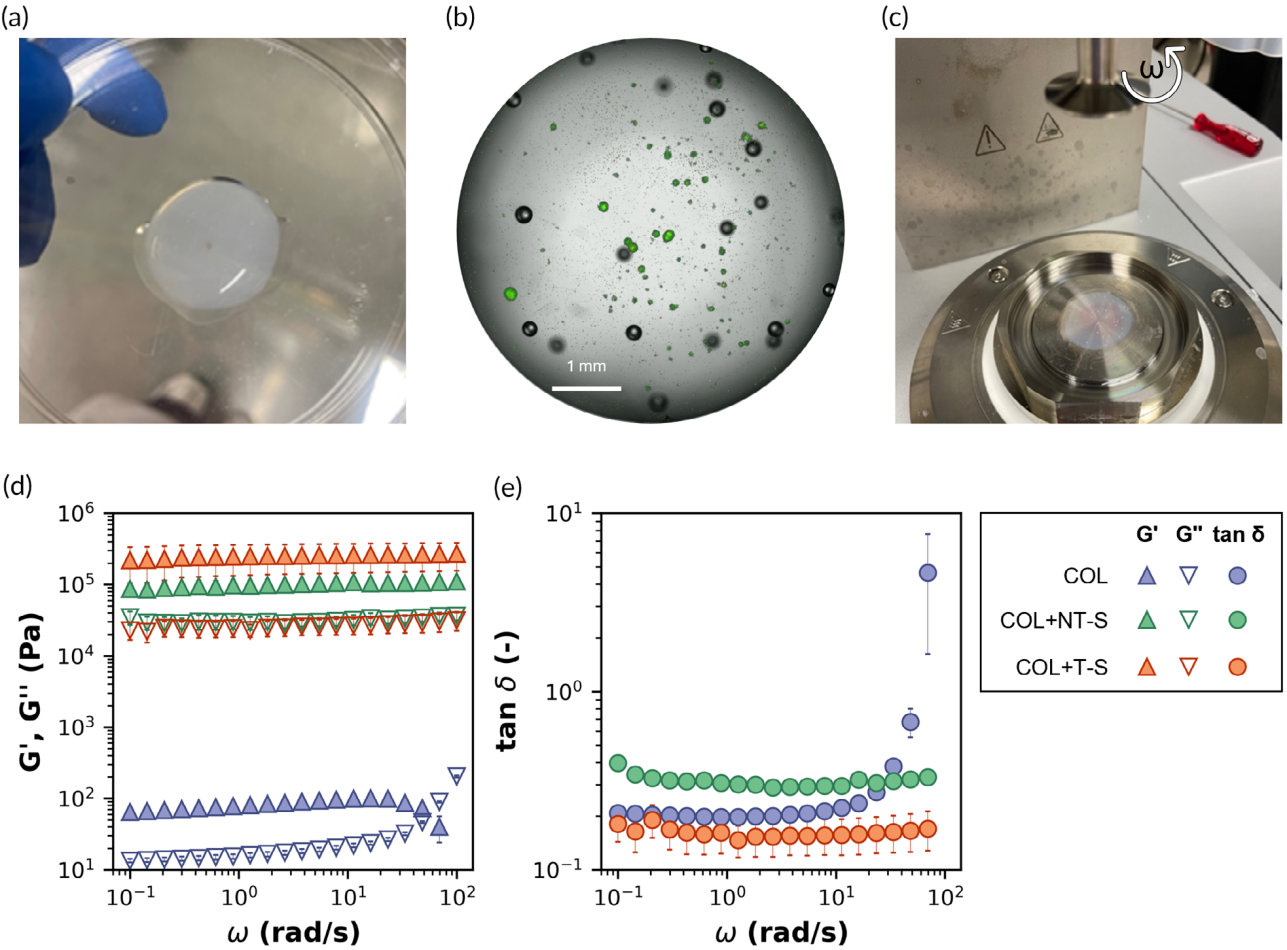
Sample preparation and viscoelastic properties of FP‐MSC (NT‐S) and MDA‐MB‐231‐GFP (T‐S) spheroids using collagen‐based hydrogel (COL) as supporting material. (a) Representative image of the COL hydrogel disk after gelation, embedding cellular spheroids prior to rheological measurements. (b) Representative bright‐field/fluorescence image showing GFP‐positive T‐S distribution within COL. Scale bar: 1 mm. (c) Rheometry setup: COL hydrogel with embedded spheroids loaded in a plate–plate geometry for oscillatory testing. (d) Viscous (*G*′, Pa) and elastic (*G*″, Pa) moduli as function of angular frequency (*ω*, rad/s) for collagenous hydrogel without and with spheroids non‐tumoral or tumoral (blue for bare supporting hydrogel (COL, *n* = 3), green with non‐tumoral spheroids (COL + NT‐S, *n* = 2) and orange with tumoral spheroids (COL + T‐S, *n* = 7)), represented by upward triangles and downward triangles, respectively. (e) Tan δ as function of angular frequency (*ω*, rad/s) for collagen‐based hydrogel without and with spheroids, represented by circles.

Indeed, tan δ (*i.e*., the ratio between *G*″ and *G*′) was analyzed for providing additional insights into the viscoelastic behavior of the system and a more comprehensive characterization of the biomechanical fidelity of the *in vitro* model. Figure 2e shows tan δ as function of ω, where biomaterial alone is represented in blue circle, COL+NT‐S in green and COL+T‐S in orange. For the supporting material, tan δ remains nearly constant at approximately 0.20 for *ω*<10^1^ rad/s. However, for *ω*>10^1^ rad/s, tan δ starts increasing, reaching a final value of approximately 4.64, indicating the transition from solid‐like to liquid‐like behavior. In contrast, for the COL+NT‐S, tan δ remains constant at approximately 0.30 across the entire frequency range and, similarly, for the COL+T‐S, it stays nearly constant at around 0.15. Consequently, tumoral spheroids exhibited a more pronounced solid‐like behavior compared to their NT counterparts.

To gain deeper insight into the biomechanics of the *in vitro* 3D models, *E* was evaluated for all tested systems. The *E* values were obtained by considering the *G*′ value at ω∼1 rad/s and applying the relationship *E* = 2*G*′(1 + *ν*), where *ν* is the Poisson ratio, set to 0.5. In particular, for incompressible materials such as fat, *ν* is typically assumed to be 0.5. Specifically, for fat pad tissue, *ν*≈0.475, while for breast fat, it is reported around 0.49.^35^ In contrast, cancerous tissues generally exhibit lower Poisson's ratios and *ν* ranges between 0.2 and 0.45 for most breast cancer tissues, depending on the subtype. The most common forms of breast cancer typically show values around 0.3–0.35, whereas triple negative breast cancer is considered nearly incompressible, with ν≈0.5.^36^, ^37^ As a result, the calculated *E* values are 2.34 ± 0.16 × 10^2^ Pa for supporting hydrogel alone, 2.91 ± 0.55 × 10^5^ Pa for COL+NT‐S and 7.36 ± 0.44 × 10^5^ Pa for COL+T‐S. These values confirm the significant difference in stiffness (three orders of magnitude larger) when spheroids are integrated, with tumoral spheroids exhibiting a much stiffer behavior compared to their healthy counterparts. Such mechanical alterations are typically associated with the ability of cancer cells, especially those in more aggressive phenotypes, to remodel the ECM, including increased collagen crosslinking and altered ECM composition, which collectively contribute to tumor rigidity increasing their contractile forces as well as promote their migration and invasive capabilities.^38^, ^39^, ^40^ These enhanced mechanical properties of MDA‐MB‐231‐GFP cancerous spheroids reflects this remodeling valuable insights into the altered biomechanical behavior of cancer cells and their role in metastasis.^41^

### 3.3 *Ex vivo* tissues biomechanics

To benchmark against *in vitro* 3D models, *ex vivo* mammary gland fat pad (healthy) and MDA‐MB‐231 tumor‐bearing tissues were excised (Figure 3a) and standardized with 5‐mm punches (Figure 3b,c). To assess the reliability of the *in vitro* models, the biomechanical properties of *ex vivo* healthy and tumoral tissues were investigated through rheological analyses and compression analyses. Figure 3d presents the rheological outcomes for healthy and tumoral tissues, highlighting key differences in their viscoelastic behavior in terms of *G*′ and *G*″ (represented by upward and downward triangles, respectively) as function of ω. As observed in the *in vitro* 3D models, the tumoral tissues (orange symbols) are stiffer than the healthy counterpart (green symbols), with *G*′ values of 2.38 ± 1.04 × 10^5^ Pa and 2.45 ± 0.93 × 10^4^ Pa at *ω*∼1 rad/s, respectively. Both systems exhibit solid‐like behavior, with *G*′ consistently higher than *G*″, specifically tumoral tissues are approximately 10 times stiffer than their healthy counterparts.

**FIGURE 3:**
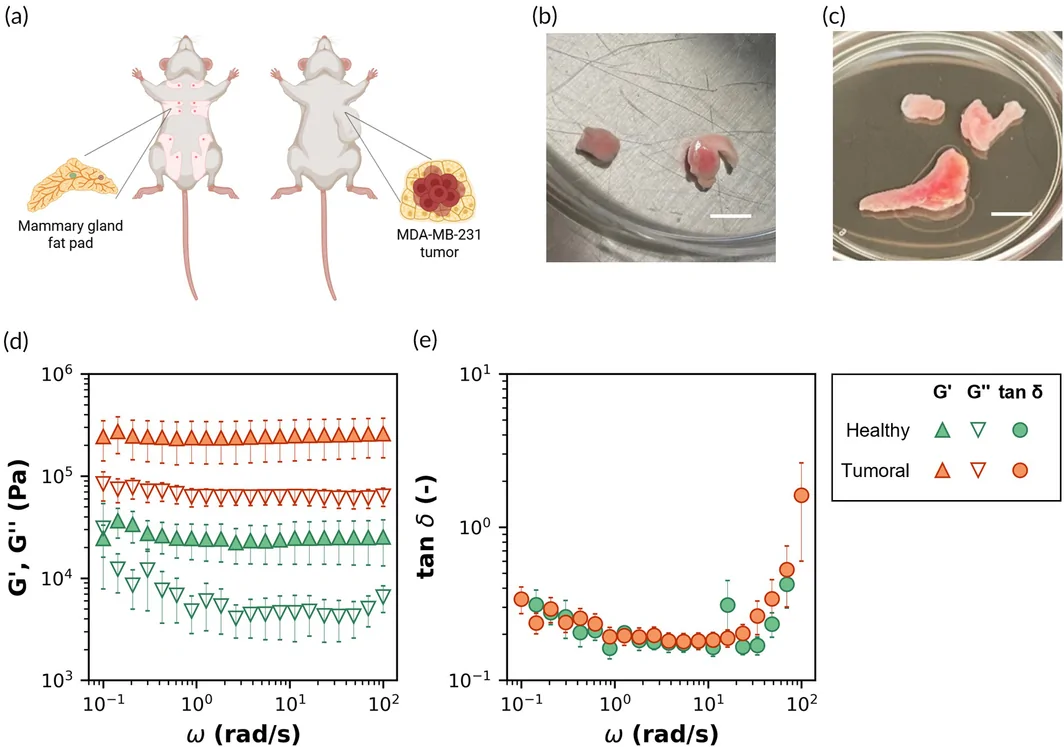
Extraction, sampling and viscoelastic properties of healthy and tumoral tissues. (a) Schematic of tissues extraction: Mammary gland fat pad used as healthy control (left) and MDA‐MB‐231 tumor‐bearing tissue used as tumoral condition (right). (b) Representative healthy explants after resection and cored with a 5‐mm biopsy punch. Scale bar: 5 mm. (c) Representative tumoral explants excised from mice bearing MDA‐MB‐231 lesion after resection and cored with a 5‐mm biopsy punch. Scale bar: 5 mm. (d) Viscous (*G*′, Pa) and elastic (*G*″, Pa) moduli as function of angular frequency (*ω*, rad/s) for healthy and tumoral tissues (green and orange symbols, respectively), represented by upward triangles and downward triangles, respectively. (e) Tan δ as function of angular frequency (*ω*, rad/s) for healthy and tumoral tissues (green and orange circles, respectively). *N* = 10 for healthy tissue and *n* = 9 for tumoral tissue.

Figure 3e further supports this finding by displaying the evolution of tan δ as a function of *ω*. Unlike the *in vitro* model, where tan δ for the healthy system was higher than for the tumoral system, here the values are quite similar for tissues regardless of pathology (0.16 vs. 0.19 for healthy and tumoral tissue, respectively) for ω<10^1^ rad/s. At higher frequencies, tan δ increases up to approximately 1.61.

Additionally, *E* was evaluated for all tested systems by considering the *G*′ value at ω∼1 rad/s and assuming ν≈0.49 for breast fat^35^ and 0.37 for tumoral tissue.^36^, ^37^ Healthy tissues exhibit an *E* value of 7.30 ± 2.77 × 10^4^ Pa, whereas tumoral tissues display a value one order of magnitude higher (6.43 ± 2.97 × 10^5^ Pa). This trend closely aligns with the developed *in vitro* 3D model results, further confirming the biomechanical relevance of the *in vitro* approach. Interestingly, the variability in the results is more pronounced in the *ex vivo* samples, particularly for tumoral tissues, and this aspect will be further clarified in the following section.

To validate the biomechanical findings on *ex vivo* samples, *E* values were also obtained using a biological compressor for both NT and tumoral tissues. Specifically, the Hertz model was employed to calculate *E* from the slope of the stress–strain (σ–ε) curve, considering only strains below 10% to ensure the analysis remained within the linear elastic regime.^42^ As shown in Figure 4a, tumoral tissues (shown in orange) exhibit an *E* value of 5.22 ± 0.62 × 10^4^ Pa, while their healthy counterpart (in green) shows a lower value of 3.68 ± 0.38 × 10^4^ Pa. This indicates that tumor tissue is approximately 1.42 times stiffer, consistent with the rheological outcomes observed for both the optimized *in vitro* 3D models and the *ex vivo* samples. Additionally, the variability observed in the compression measurements is lower than that in standard rheology (Figure 4b), likely due to the smaller variation in the applied normal force (*F*_N_), while maintaining a constant cross‐sectional area.

**FIGURE 4:**
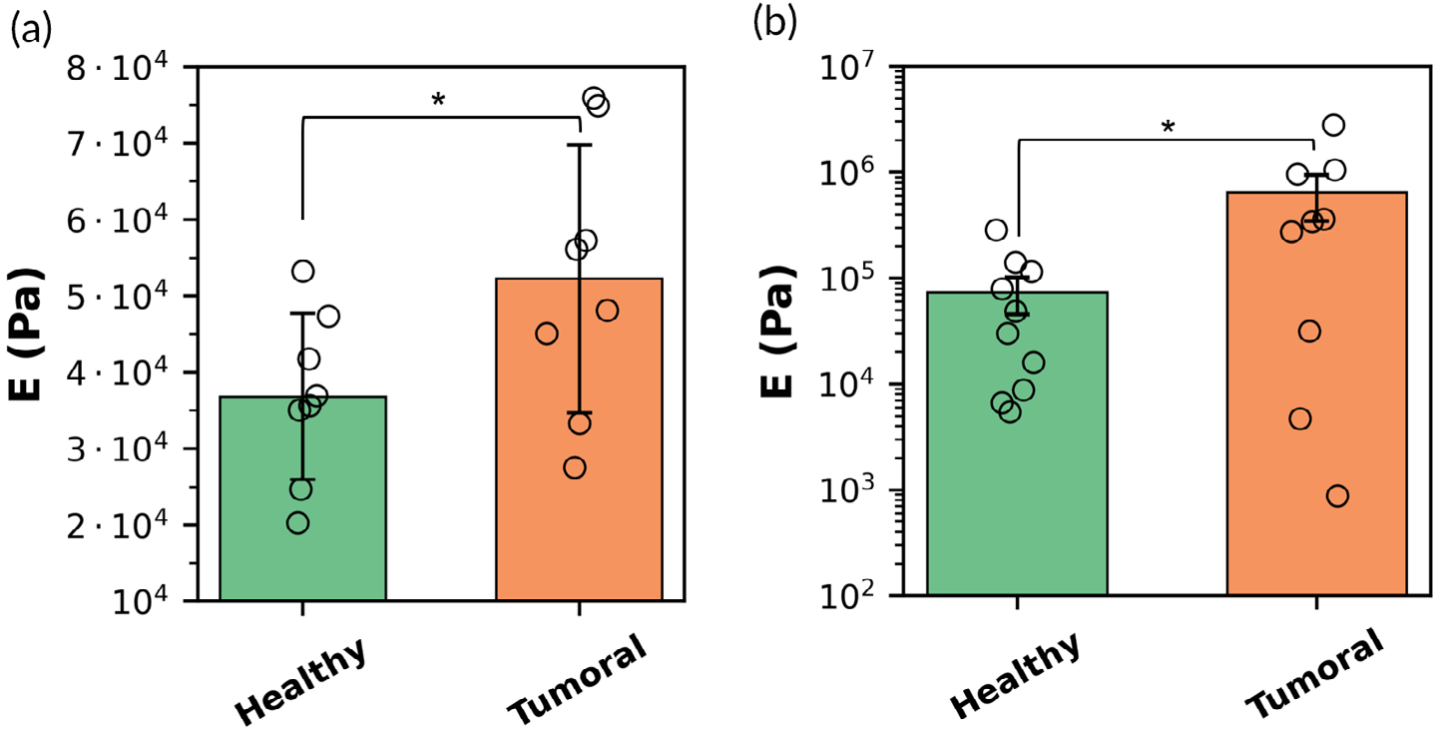
Mechanical evaluation of *ex vivo* tissues. Comparison of the Young's modulus (*E*) for non‐tumoral and tumoral tissues (green and orange, respectively) in (a) compression tests and (b) standard rheology. Statistical significance of the results: *p<0.05.

Notably, depending on the pathological status, biological samples may exhibit either force‐softening (healthy tissues) or force‐stiffening (tumoral tissue) behaviors. Such non‐linear responses can introduce artifacts into the characterization process, compromising a direct correlation between the viscoelastic properties of ECM and critical cellular behaviors, such as migration, proliferation, or differentiation.^43^, ^44^ However, when accounting for the normal force applied, a very good agreement between mechanical outcomes can be achieved. In fact, the discrepancy in the absolute values of *E* between rheological and compression‐based measurements (i.e., 7.30 ± 2.77 × 10^4^ Pa vs 3.68 ± 0.38 × 10^4^ Pa for healthy tissues, respectively; and 6.43 ± 2.97 × 10^5^ Pa vs. 5.22 ± 0.62 × 10^4^ for tumoral tissues, respectively) can be attributed to differences in the applied normal force. During standard rheological testing, samples were intentionally compressed to ensure full contact between the biological tissue and the rheometer plates (*i.e*., *d*_tissues_∼*d*_tool_) thereby minimizing measurement artifacts.^43^, ^44^ Due to sample variability, different normal forces were applied along the tissue surface, resulting in variations in the measurement gap, typically ranging from 0.1 to 0.7 mm for healthy tissues and from 0.3 to 1.0 mm for tumoral tissues. Figure 5 display the elastic (*G*′, solid upward triangles) and viscous (*G*″, open downward triangles) moduli for both healthy and tumoral tissues (Figure 5a,c, respectively), plotted as a function of the applied *F*_N_ while keeping the cross‐sectional area constant and approximated by the tool area (*A* = π*R* ^2^~491 mm^2^). In all conditions, *G*′ consistently exceeds *G*″, confirming the predominantly elastic nature of the tissues. Interestingly, healthy tissues (Figure 5a) exhibit a force‐softening response, with moduli decreasing by at least one order of magnitude as the *F*_N_ increases. In contrast, tumoral tissues (Figure 5) show a force‐stiffening behavior, whereby higher force leads to increased moduli.

**FIGURE 5:**
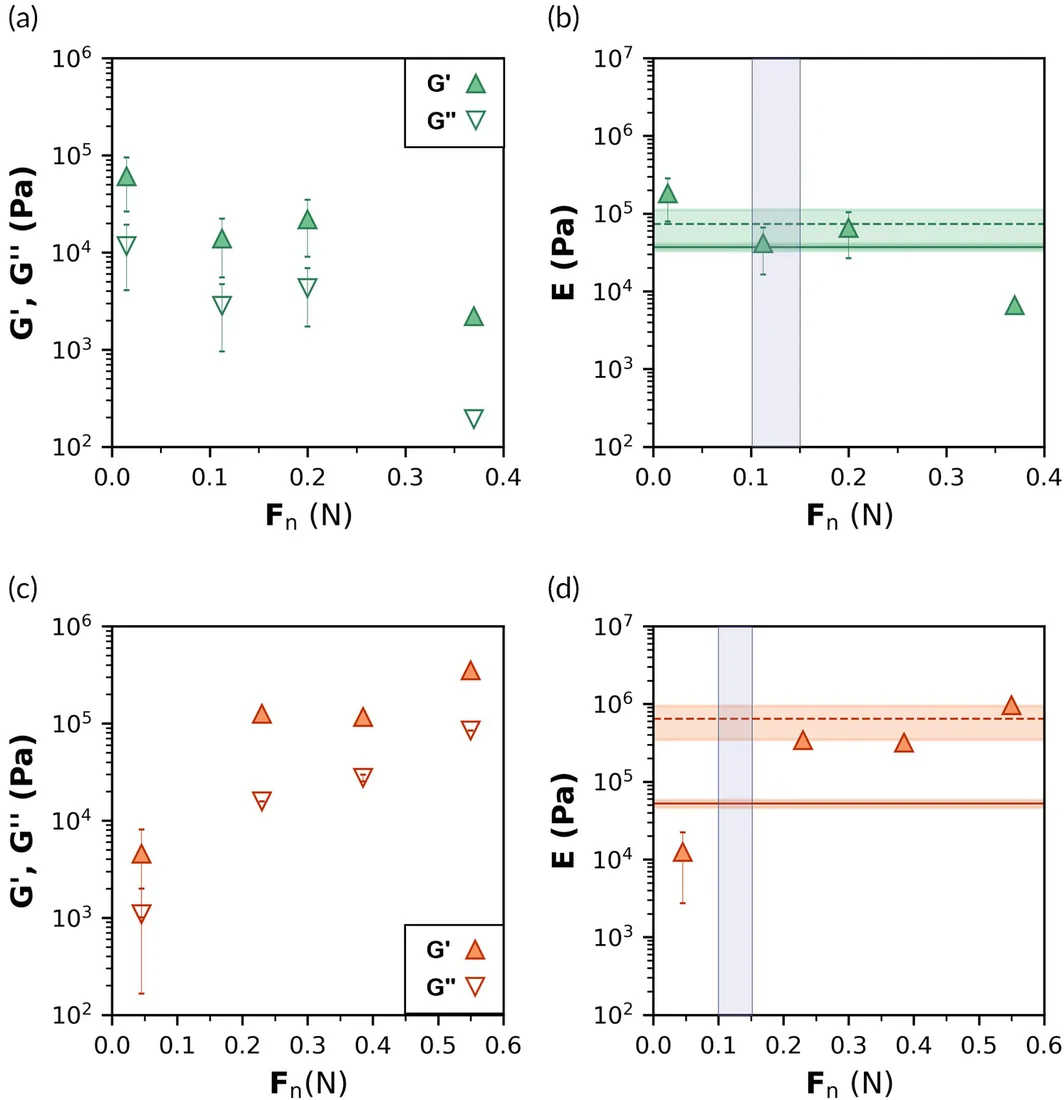
Viscoelastic properties of healthy and tumoral tissues as a function of applied normal force (*F*_N_). (a), (c) Elastic (*G*′, Pa) and viscous (*G*″, Pa) moduli plotted against applied normal force (*F*_N_, N) for healthy (green triangles) and tumoral (orange triangles) tissues, represented by upward triangles and downward triangles, respectively. (b), (d) Comparison of the Young's modulus (*E*) for healthy (green) and tumoral (orange) tissues, estimated using the relation *E* = 2*G*′(1 + *ν*), with *G*′ evaluated at ω≈1 rad/s. In both panels, the mean *E* values obtained from standard rheological measurements (dotted line) and from biological compression tests (solid line) are indicated.

To better compare the results of standard rheology with those from the compression assay, Figure 5b,d shows the corresponding *E*, calculated by assuming the previously discussed *ν* (0.49 and 0.37 for healthy and tumoral tissues, respectively), plotted as a function of *F*_N_. In both plots, the average *E* value obtained via standard rheology is marked by a dotted line (7.30 × 10^4^ Pa and 6.43 × 10^5^ Pa for healthy and tumoral tissues, respectively). During compression testing, the *F*_*N*_ reached for strain ε <10% was approximately 0.1–0.15 N. Within this *F*_N_ range, the predicted *E* values from rheology closely match those measured *via* compression (3.68 × 10^4^ Pa and 5.22 × 10^4^ Pa for healthy and tumoral tissues, respectively), indicated by solid lines. This agreement allows for a more reliable comparison of tissue elasticity across the two methods. Notably, the discrepancy observed between rheology and compression‐based values, more pronounced in tumoral tissues, is effectively resolved when actual *F*_N_ is accounted for, demonstrating strong consistency between the datasets. This highlights the importance of comparing mechanical properties within similar normal force regimes to ensure consistency between characterization methods.

## 4 DISCUSSION

Understanding and quantifying tumor biomechanics is gaining momentum as a critical dimension of cancer research, with growing evidence that mechanical alterations within the tumor microenvironment actively influence tumor progression, metastasis, and resistance to therapy. Despite this recognized importance, tools capable of capturing and comparing tumor mechanical properties across biological systems remain limited. In this context, the platform presented in this study offers a technologically robust and biologically relevant solution, bridging 3D *in vitro* spheroids with *ex vivo* tissue analysis through a unified rheological and compression‐based approach.

Our findings confirm that breast cancer spheroids derived from MDA‐MB‐231 cells exhibit significantly higher stiffness and nonlinear force‐dependent behavior compared to their NT counterparts, reflecting ECM remodeling associated with aggressive phenotypes. Moreover, the choice of a collagen‐based hydrogel ensured that the supporting matrix did not mask spheroid mechanics while maintaining a biomimetic environment. The difference between conditions was preserved across *in vitro* and *ex vivo* models, with tumor tissues showing elevated storage modulus (*G*′) and distinct nonlinear behavior under load, both signatures of ECM remodeling and increased cellular contractility. The consistency between *in vitro* and *ex vivo* measurements across force regimes supports the mechanical fidelity and transferability of our *in vitro* system and validates its potential as a preclinical platform for studying or testing mechano‐sensitive tumors and stiffness‐adapted therapies. While further validation is required, this work lays the groundwork for integrating mechanical parameters into diagnostic workflows and therapeutic planning.

Importantly, this study highlights the complementary nature of rheological and compression‐based assessments. Technologically, the choice of rotational rheometry as a central characterization tool marks a critical advantage. While compression testing remains standard in tissue mechanics, it often oversimplifies tissue mechanics by focusing solely on elastic properties,^45^ typically through the application of the Hertz model. In contrast, rheology provides a richer, frequency‐dependent profile of viscoelastic behavior, critical for capturing the dynamic nature of tumor response. The combination of rheological and mechanical compression techniques highlights the strengths and complementary nature of these techniques, with rheology providing a more comprehensive understanding of material behavior at both the cellular and tissue levels. The agreement between these methods within specific normal force windows emphasizes the need for careful comparison of mechanical properties under consistent conditions. Our data shows that force‐sensitive shifts in stiffness can significantly affect measurements and should be standardized or accounted for in future biomechanical profiling.

From a translational standpoint, the observed mechanical differences may have direct implications for therapy design and diagnostics. Increased tumor stiffness has been shown to hinder drug diffusion, impair immune cell infiltration, and activate mechanotransduction pathways that promote chemoresistance.^5^, ^6^ By reproducing this stiffness landscape *in vitro* and aligning it with *ex vivo* profiles, our system enables a more predictive evaluation of mechanically responsive therapies. Moreover, the mechanical fingerprinting of tumor models could support the development of diagnostic criteria based on physical properties, especially when integrated with imaging techniques or functional assays. Moreover, because breast cancer biology and stromal responses can be modulated by sex‐related factors and donor‐to‐donor variability, future validation aimed to strengthen the translational framing and interpretability of our study will benefit from considering sex as a biological variable and primary cells from different human subjects. Especially hormone‐dependent signaling like estrogen receptor pathways can modulate stromal cell behavior and ECM remodeling, which may ultimately influence the magnitude and dynamics of bulk mechanical readouts.^46^ In parallel, validation across a broader set of primary cell donors will be essential to discriminate whether baseline phenotype, inflammatory priming, age‐ and clinical background–related differences can introduce additional dispersion in viscoelastic metrics.

Critically, the reproducibility and scalability of our platform make it suitable not only for academic investigation but also for industrial applications in drug screening and biomaterials testing. The use of standardized hydrogel scaffolds, controlled spheroid assembly, and quantitative mechanical output positions this approach as a translatable solution for early‐stage therapeutic validation or personalized medicine pipelines. While further work is needed to expand the system to other tumor subtypes and integrate dynamic environmental conditions (*e.g*., perfusion, immune components), the current framework offers a solid starting point for embedding biomechanical profiling into preclinical workflows.^47^ Although bulk metrics provide standardization and throughput for cross‐condition discrimination, they alone are not sufficient to uniquely describe the mechanical microenvironment experienced by individual cells, which can vary within the tumor due to spatial heterogenicity in ECM density, local architecture, and remodeling. Despite this limitation, bulk mechanics remain valuable for standardized comparisons across model conditions, benchmarking mechanically distinct tumor models using a single quantitative scale, and capturing global load‐bearing behavior relevant to macroscopic deformation and transport. By contrast, microenvironmental mechanics measured at the cellular scale can be indicative of local stiffness gradients and heterogeneity, directly relevant to mechanotransduction. Other spatial measurement approaches such as AFM couple with indentation mapping, microrheology, and traction‐based often require careful model assumptions (*e.g*., contact mechanics, boundary conditions) and may be sensitive to local geometry and sampling location.^21^, ^22^, ^23^, ^24^, ^25^, ^26^, ^27^ Therefore, bulk and cell‐scale mechanics should be considered complementary rather than competing descriptors. While our study focuses on establishing reproducible bulk viscoelastic metrics across breast cancer spheroid models, future work integrating different cell populations to recreating tissue heterogenicity and spatial transcriptomics along with micromechanical mapping could better connect stiffness heterogeneity to specific cellular phenotypes including invasion, stem‐like programs, and therapy response. In summary, the platform presented here combines engineered biological models with precision mechanical analysis, offering a scalable and reproducible tool for translational breast cancer research. By bridging tissue‐level biomechanics and experimental oncology, this approach opens new directions for mechanics‐informed diagnostics, therapy design, and patient‐specific modeling.

## 5 CONCLUSION

This proof‐of‐concept study introduces a scalable platform for biomechanical profiling of breast cancer using a multimodal strategy that bridges 3D *in vitro* spheroids and preliminary *ex vivo* tissue mechanics, providing insights into viscoelastic tumor behavior, an often‐overlooked dimension with growing clinical relevance.

The ability to reproduce and compare mechanical signatures across biological systems supports applications in drug screening and mechanics‐informed therapy design and physical biomarker discovery, aiming to improve drug delivery and treatment efficacy in cancer patients. Furthermore, given the importance of ECM stiffness in cancer biology as a predictor of therapeutic resistance and disease progression, our platform offers a reproducible framework for embedding mechanical evaluation into experimental and translational oncology workflows.

Future developments will focus on expanding this strategy to larger *ex vivo* cohorts to validate robustness, broaden biological complexity by incorporating additional cell populations and additional tumor types, incorporating dynamic cues (*e.g*., flow, immune components), and exploring integration with diagnostic imaging and patient‐specific models.

## AUTHOR CONTRIBUTIONS

**Federica Banche‐Niclot:** Writing – original draft; validation; formal analysis; data curation; visualization; methodology; conceptualization; software. **Francesca Taraballi:** Funding acquisition; writing – review and editing; project administration; supervision; resources. **Paola de Paolis:** Investigation; formal analysis. **Valerio Di Palo:** Investigation; formal analysis. **Rosalia Ferraro:** Writing – original draft; validation; visualization; formal analysis; data curation; methodology; conceptualization; software. **Sergio Caserta:** Funding acquisition; writing – review and editing; project administration; supervision; resources.

## CONFLICT OF INTEREST STATEMENT

The authors declare that they have no known competing financial interests or personal relationships that could have appeared to influence the work reported in this paper.

## Supporting information


**Figure S1.** Representative bright‐field images of FP‐MSC (a)–(c) and MDA‐MB‐231‐GFP (d)–(f) spheroids used for size evaluation. Scale bar: 100 μm.

## Data Availability

The data that support the findings of this study are available from the corresponding author, F.T. and S.C., upon reasonable request.
