# Supplementary material for: Mechanical fingerprints in breast cancer research: A multimodal experimental approach
Source: Bioeng Transl Med. 2026 May 8;11(5):e70148. doi: 10.1002/btm2.70148 (PMC13636252; doi:10.1002/btm2.70148)
Supplement: Supplementary file 1 [file BTM2-11-e70148-s001.docx]

**Mechanical Fingerprints in Breast Cancer Research: A Multimodal Experimental Approach**

**Figure S1**. Representative bright-field images of FP-MSC (**a-c**) and MDA-MB-231-GFP (**d-f**) spheroids used for size evaluation. Scale bar: 100 μm.
